# CAR-T cells based on a TCR mimic nanobody targeting HPV16 E6 exhibit antitumor activity against cervical cancer

**DOI:** 10.1016/j.omton.2024.200892

**Published:** 2024-10-09

**Authors:** Zhijian Duan, Dan Li, Nan Li, Shaoli Lin, Hua Ren, Jessica Hong, Christian S. Hinrichs, Mitchell Ho

**Affiliations:** 1 Antibody Engineering Program, Center for Cancer Research, National Cancer Institute, National Institutes of Health, Bethesda, MD 20892, USA; 2 Laboratory of Molecular Biology, Center for Cancer Research, National Cancer Institute, National Institutes of Health, Bethesda, MD 20892, USA; 3 Rutgers Cancer Institute of New Jersey, New Brunswick, NJ 08901, USA

**Keywords:** HPV16+, E6-MHC, TCR mimic, nanobody, CAR-T, cervical cancer

## Abstract

The E6 and E7 oncoproteins of human papillomavirus (HPV) are considered promising targets for HPV-related cancers. In this study, we evaluated novel T cell receptor mimic (TCRm) nanobodies targeting the E6_29-38_ peptide complexed with human leukocyte antigen (HLA)-A∗02:01 in the chimeric antigen receptor (CAR) format. We isolated two dromedary camel nanobodies, F5 and G9, through phage display screening. F5 bound more efficiently to the complex expressed on cells, including peptide-pulsed T2, overexpressed 293E6, and cervical cancer lines CaSki and SS4050, compared to G9. CAR-T cells based on the F5 nanobody specifically killed target cells, including 293E6, CaSki, and SS4050 *in vitro*, through activation of nuclear factor of activated T cells (NFAT) and nuclear factor κB (NF-κB) signaling. Importantly, F5 CAR-T cells inhibited the growth of CaSki and SS4050 tumor xenografts in mice. These findings demonstrate that HPV-16+ cervical cancer can be targeted by F5 nanobody-based CAR-T cells, offering a valuable alternative strategy for treating HPV-16+ malignancies.

## Graphical abstract

**Figure:**
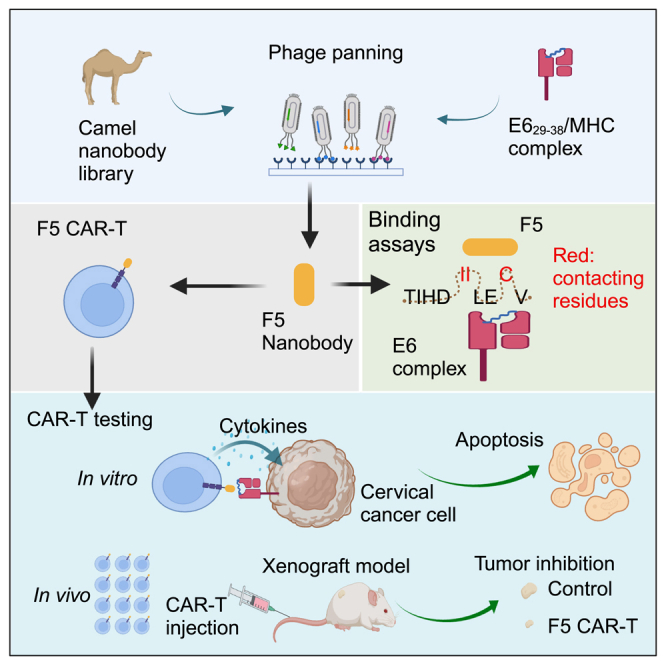

## Teaser

Ho and colleagues discovered a camel single-domain antibody, known as F5, that targets the oncogenic E6 of human papillomavirus (HPV) 16. Chimeric antigen receptor (CAR) T cells based on this nanobody showed promising efficacy in killing HPV16+ tumor lines and inhibiting tumor growth in cervical cancer xenografts in mice.

## Introduction

Current antibody-based cancer immunotherapies target a limited number of tumor-specific surface proteins. However, most oncogenic drivers are intracellular proteins that are not easily accessed by antibody-based immunotherapy.^1^^,^^2^ The only part of these oncogenic proteins accessible to the immune system is the peptide presented by the major histocompatibility complex (MHC; also known as human leukocyte antigen [HLA] in human) on the cell surface. The peptides originate from various intracellular tumor antigens, including viral oncogene products, transcription factors, oncofetal proteins, cancer-testis antigens, and neoantigens from mutated oncogenes.^1^^,^^2^^,^^3^^,^^4^ Those peptide-MHC (pMHC) complexes can be targeted by engineered T cell receptor (TCR) therapy. However, the major drawback of TCR therapy is that TCRs might be highly individualized and limited to the MHC class.^5^^,^^6^ One attractive alternative is to develop TCR-like or TCR mimic (TCRm) antibodies that recognize pMHC complexes and mimic the binding of TCRs to the complexes. TCRm antibodies could expand the range of therapeutic targets to intracellular proteins and thus have broad clinical potential. Progress has been made in the development of TCRm antibodies targeting aberrantly expressed intracellular oncogenic and tumor-associated antigens (TAAs), such as Wilms tumor 1 (WT1), gp100, MAGE-A3, Melan-A, and NY-ESO-1.^1^^,^^2^^,^^7^^,^^8^ Recent studies by Hsiue et al. and Douglass et al. described the development of TCRm antibodies that recognize the mutation-associated neoantigens derived from TP53 or KRAS.^9^^,^^10^ So far, TCRm-antibody-based therapeutics targeting WT-1 and AFP have advanced to clinical trial stages.^1^^,^^11^

Human papillomavirus (HPV) viral antigens E6 and E7 are ideal targets for TCRm antibody development because they are oncogenic and constitutively expressed by tumors but not by healthy tissues.^12^^,^^13^^,^^14^ HPV has been linked to cancers of the uterine cervix, oropharynx, anus, vulva, vagina, and penis. HPV types 16 and 18 are the most virulent high-risk genotypes and are responsible for approximately 60% and 15% of cervical cancers, respectively.^15^ Although HPV vaccines aid in the prevention of HPV-associated cancers, there are still more than 5,000 deaths caused by HPV-associated cancers each year in the US. Also, cervical cancer continues to be the second leading cause of cancer death in women aged 20–39 years.^16^ While recent progress has shown that engineered T cell therapy has demonstrated its efficacy and safety in patients with HPV-associated malignancies, especially with metastatic tumors, tumor resistance and immune escape can still lead to the ineffectiveness of the treatment in patients.^12^^,^^13^^,^^17^ In addition, widespread implementation is constrained by the need for patients’ autologous cells and sophisticated manipulation of cells in an individualized manner. Therefore, innovative treatment is still urgently needed. To develop TCRm antibodies against E6 or E7 for HPV-related cancers, the presence of neoantigens on the tumor cell surface is the foundation. In HPV16+-related epithelial cancers, E6_29-38_ or E7_11-19_ peptides complexed with HLA-A∗02:01, which is the most common allele in the White/Caucasian population, are present in tumor cells and demonstrated by several TCR cell therapy papers.^12^^,^^13^ A recent study reported a TCRm antibody (3F8) targeting E7_11-19_ in the context of HLA-A∗02:01. This antibody showed T cell-redirected cytotoxicity as a bispecific T cell engager (BiTE) with modest efficacy.^18^

The typical 8- to 14-mer peptide presented in MHC class I comprises only around 2%–3% of the amino acids in the pMHC complex and is spatially confined within the adjacent α helices of the MHC groove.^19^ The epitope described here is generally difficult to reach by conventional immunoglobulin (Ig)G-based antibodies and thus poses a challenge for TCRm antibody development. Single-domain antibodies or nanobodies include the antigen-binding variable domains of the shark immunoglobulin new antigen receptor (V_NAR_) and the camelid variable region of the heavy chain (V_H_H).^20^^,^^21^^,^^22^ We hypothesize that single-domain antibodies might be suitable for the development of TCRm antibodies because they are much smaller (around 15 kDa) than conventional antibodies and have shown a special ability to target buried epitopes in protein antigens.^21^^,^^23^^,^^24^ For instance, a shark nanobody against the lysozyme could bind to a cavity in the enzyme pocket, which might be inaccessible for conventional IgG.^23^ In addition, we recently isolated a dromedary camel nanobody D5 neutralizing Lassa virus (LASV), and D5 bound to the glycan-free hole at the apex of the virus glycoprotein trimer (GPC), which was a small and unique site.^24^ Using our dromedary camel V_H_H phage libraries, we identified two nanobodies, F5 and G9, against HLA-A∗02:01-complexed E6_29-38_, which is a well-known epitope^25^^,^^26^ and has been targeted by TCR gene-engineered T cells.^12^ F5 and G9 could recognize the E6 complex specifically in the protein form or expressed on cells. F5 chimeric antigen receptor (CAR)-T cells showed specific killing of the target cells *in vitro* and inhibited the growth of CaSki or SS4050 tumor xenografts in mice. This TCRm nanobody has potential as an immunotherapy directed against HPV-16+ malignancies.

## Results

### Identification and characterization of camel V_H_Hs F5 and G9

**Figure 1:**
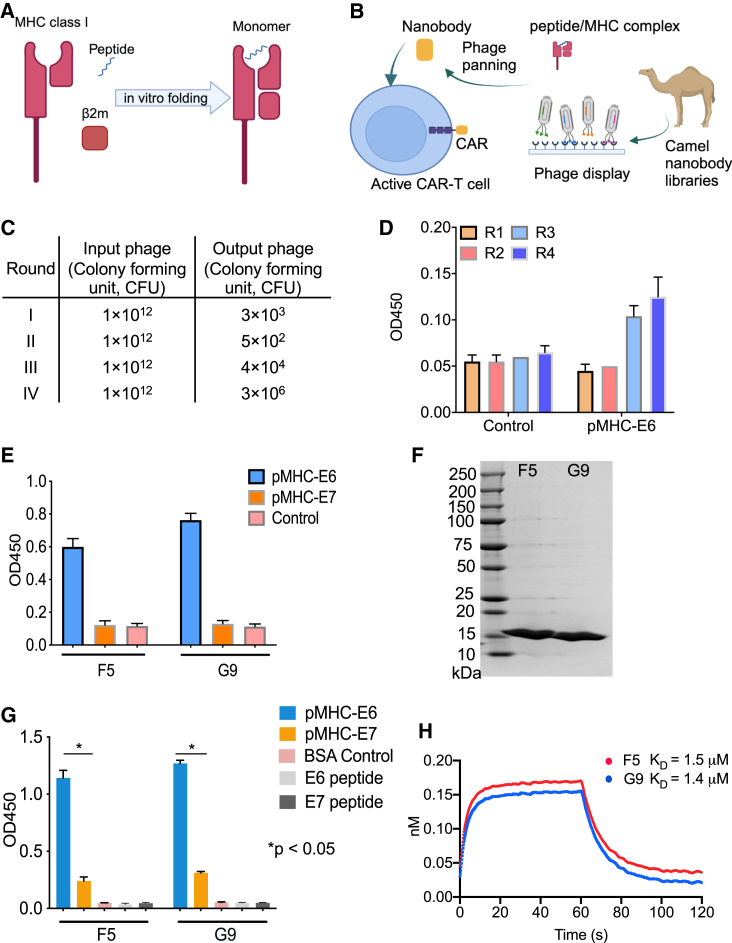
Two TCRm nanobodies, F5 and G9, were identified by phage panning from camel single-domain antibody libraries (A) Diagram of the monomer synthesis from the NIH Tetramer Core. (B) Diagram of phage panning process, nanobody discovery, and following application as CAR format. (C) The input and output of phage numbers in the four rounds of phage panning. (D) Polyclonal phage ELISA results from the panning. (E) Monoclonal phage ELISA of the two binders, F5 and G9, against the antigens. (F) SDS-PAGE gel image of the two binders, F5 and G9, from protein purification. (G) ELISA data showing the protein binding of F5 and G9 to the antigens. (H) K_D_s of F5 and G9 to pMHC-E6 complex by bio-layer interferometry (BLI) technology. ∗*p* < 0.05.

The monomers of E6_29-38_ or E7_11-19_ peptides complexed with HLA-A∗02:01 were synthesized at NIH Tetramer Core (Figure 1A). To identify nanobodies against the monomer, phage panning was carried out using camel single-domain phage display libraries constructed from six camels, three males and three females, with ages ranging from 3 months to 20 years (Figure 1B). After four rounds of panning, there was an increased phage output (Figure 1C) and about 3-fold enrichment of eluted phage colonies by polyclonal phage ELISA (Figure 1D). At the end of the fourth round of panning, 20 individual clones were identified to bind the E6 monomer protein by the monoclonal phage ELISA, and eight unique binders were confirmed by subsequent sequencing. Eventually, two camel V_H_Hs binders, F5 and G9, were identified that showed specific binding to the E6 monomer on monoclonal phage and protein ELISA (Figures 1E–1G). An Octet analysis was performed to measure the binding affinity against the E6 monomer. The K_D_ values of F5 and G9 were around 1 μm, which is close to the affinity of most TCRs binding to their target complexes (Figure 1H).^27^

### Binding of F5 and G9 to the cells

**Figure 2:**
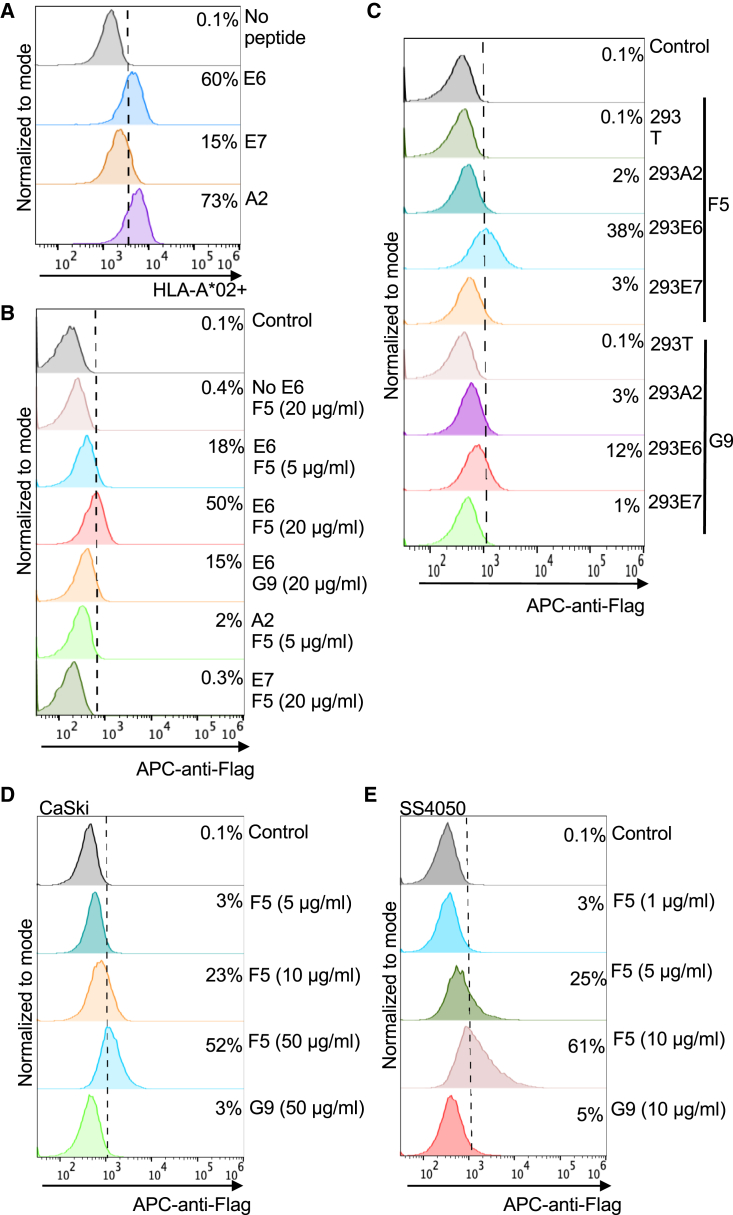
F5 and G9 had different binding to the complexes expressed on cells (A and B) Peptide pulsing on T2 cells. Peptides (50 μM) were pulsed on 1 million T2 cells overnight, and the cells were stained with anti-HLA antibody for FACS (A). Cells were also stained with the nanobodies to measure the binding by FACS (B). (C) The binding of F5 and G9 to the complexes on 293T, 293A2, 293E6, and 293E7 cells by FACS. (D and E) The binding of the nanobodies on tumor cell lines CaSki (D) and SCC90 (E).

We further validated the binding specificity of nanobodies to E6-MHC on the cell surface of human T2 lymphoblast cells. T2 cells are deficient in the transporter associated with antigen processing (TAP) and express unstable empty HLA molecules on the surface. Adding the peptide and beta-2-microglobulin stabilizes the HLA molecule on the cell surface by forming a complex, which can be measured by fluorescence-activated cell signaling (FACS) with an anti-HLA-A∗02 antibody. The E6, E7, and A2 (influenza virus) peptides were pulsed on T2 cells successfully (Figure 2A). F5 and G9 stained the cell surfaces of those T2 cells that were pulsed with the target E6 peptide but not the ones pulsed with the E7 and flu-A2 peptides (Figure 2B). G9 had less binding to the complex than F5 at the same concentration (20 μg/mL) (15% vs. 50%), suggesting the weaker binding ability of G9. To further validate the binding specificity of F5 and G9, 293 cells expressing the E6 or E7 complex were used. F5 and G9 bound 293E6 more specifically than 293E7, 293A2, and 293T cell controls. F5 showed a higher binding signal to 293E6 cells than G9 (Figure 2C). Next, we investigated whether F5 could recognize a naturally processed E6 complex on the surface of cancer cells. Among the HPV16+ cancer cell lines tested (CaSki, SS4050, and SCC90), F5 showed high binding to CaSki and SS4050 cells in a dose-dependent manner, while G9 showed weak binding (Figures 2D and 2E). SS4050 showed a much higher signal than CaSki (25% vs. 3%) at 5 μg/mL of F5, indicating the higher antigen expression level in SS4050 cells (Figures 2D and 2E). The binding on SCC90 was shallow (data not shown).

### No off-target binding of nanobody F5

To investigate the potential off-target reactivity of nanobody F5, we searched peptide databases, including PeptideAtlas and UniProt, but did not find a similar peptide in humans. Using NCBI/Blast, the closest human sequence identified was TIHDINSSLILE (12-mer) from the inaD-like protein 799-810. This sequence has 67% identity with the 10-mer E6_29-38_ (TIHDIILECV). To predict the peptide binding to the HLA allele using NetMHCpan, the identified inaD-like protein peptide was associated with a very low prediction score (0.0001870) for binding to HLA-A∗02:01, whereas the score for E6_29-38_ was significantly higher (0.3267180). Although it is very unlikely that the identified inaD-like protein peptide could bind to HLA-A∗02:01, we decided to conduct a pulsing assay with this peptide. The results indicated that this peptide indeed could not be loaded onto T2 cells, and F5 staining showed minimal background binding in this group (Figure S1). Therefore, it is unlikely that F5 could recognize other similar peptide sequences in humans.

Another concern about the cross-reactivity is the binding to the same peptide presented in different HLA alleles, which actually might be beneficial for a broader population. It has been validated that the following HPV E6 peptides are coupled with different HLA alleles: E6_29-38_-HLA-A∗02:01(dominant in the White population), E6_93-101_-HLA∗11:01 (dominant in the East Asian population), and E6_49-57_-HLA-A∗24:02 (dominant in the Southeastern population).^28^ Currently, there is no direct evidence that E6_29-38_ can be presented in all these alleles yet, aside from HLA-A∗02:01. However, it has been predicted that E6_29-39_ might be presented on HLA-A∗26:01,^28^ although this has not yet been validated by analyzing patient samples using mass spectrometry. To address this question, we recently purchased monomers from a commercial source: HLA-A∗02:01 (positive control), HLA-A∗11:01, HLA-A∗24:02, and HLA-A∗26:01. According to the company’s instructions, these monomers can be mixed with a peptide to generate peptide/MHC complexes. ELISA results with anti-HLA-A∗02(BB7.2) and anti-HLA class I (W6/32) antibodies showed comparable antigen coating on the plate among different monomers (Figure S2B). F5 at different concentrations (5, 10, 20, and 40 μg/mL) showed specific binding to the groups of the E6 monomer from the NIH Tetramer Core and the commercial HLA-A∗02:01 + E6 peptide compared with controls (Figure S2A). F5 at concentrations of 5, 10, and 20 μg/mL did not show statistically significant binding to HLA-A∗26:01 + E6 compared with HLA-A∗26:01 alone (*p* > 0.05) (Figure S2A). However, F5 at 40 μg/mL did show some difference (*p* = 0.04). Since there was no dose-dependent binding of F5 to HLA-A∗26:01 + E6, this binding difference at high concentrations was not convincing. F5 also did not show different binding signals to the monomers of HLA-A∗24:02 or HLA-A∗11:01 compared with no peptide loading (Figure S2A).

### The residues of E6 peptide involved in F5 and G9 binding to the complex

**Figure 3:**
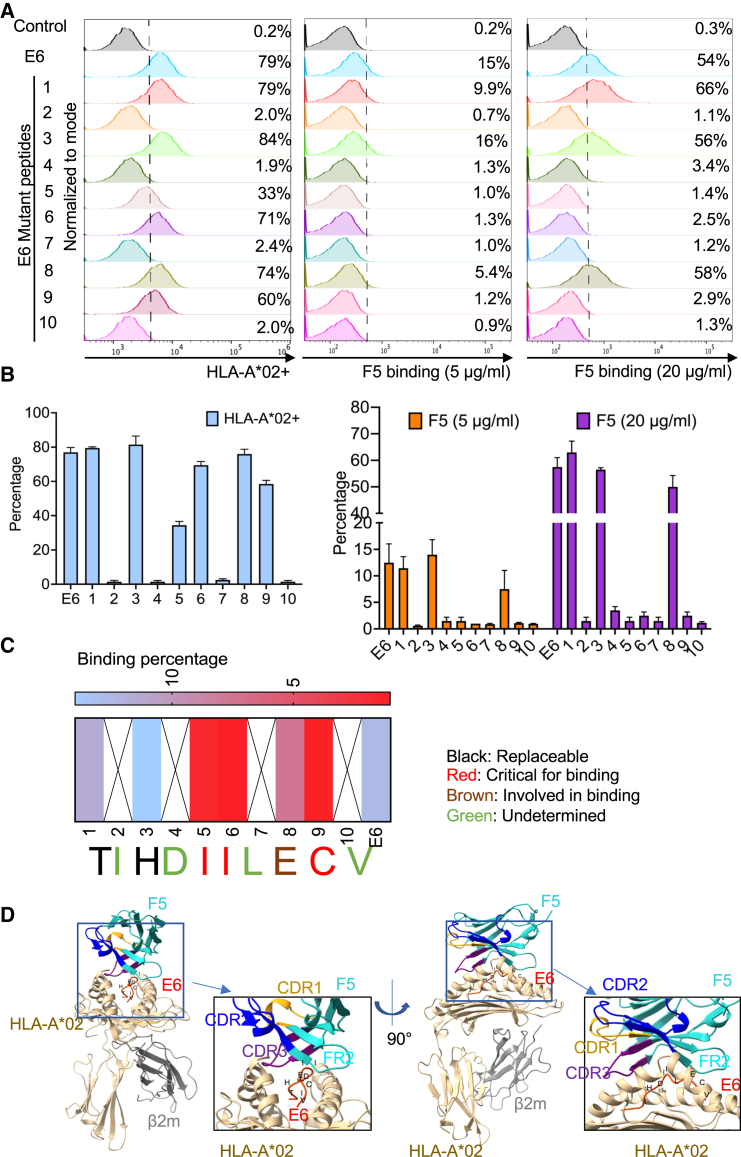
The C-terminal residues of E6 peptide were involved in F5 binding to the complex (A–C) T2 cells were pulsed with E6 and 10 mutated peptides, which have an individual single mutation to alanine at each position (50 μM), and then examined by FACS for the expression of the complex and F5 binding at 5 and 20 μg/mL. (D) Docking of F5 to the pMHC-E6 by i-TASSER and ClusPro.

To determine the residues on the peptide that are involved in the binding of F5 and G9 to the complex, an alanine scanning of the E6 peptide was performed. Each amino acid of the E6_29-38_ peptide (TIHDIILECV) was mutated to alanine and synthesized with a high purity (Table S1). The 10 mutated peptides were pulsed on T2 cells, and the binding of F5 to the complex was measured by FACS. Mutations in sites 2, 4, 7, and 9 had low expression levels of complexes measured by anti-HLA antibody staining with FACS (Figures 3A and 3B), suggesting that those four sites are involved in the peptide binding to the HLA molecule and are essential for complex formation. Therefore, their role in F5 binding could not be determined here. Mutations 1 and 3 did not affect the binding of F5, suggesting that those two residues are replaceable and might not be involved in binding. Mutations 5, 6, and 9 resulted in a complete loss of F5 binding to the complex, indicating that those residues are critical for binding. Mutation 8 led to decreased binding of F5 to the complex, which was more evident at low F5 concentrations (5 μg/mL). It is possible that although residue 8 is involved in binding, it plays a less critical role than residues 5, 6, and 9 (Figures 3A–3C). The mutation binding pattern of G9 is similar to that of F5, but it exhibited some differences (Figure S3). G9 did not bind to the complexes with mutations 5, 6, and 9 like F5 did. Additionally, residue 8 was replaceable for G9 binding with residues 1 and 3, which differs from F5.

We further predicted the binding of V_H_Hs to the complex using online tools. The structures of V_H_Hs and the complex were predicted by iTASSER,^29^ and antibody-antigen docking was performed with ClusPro (Figure 3D).^30^ Predicted models showed that the FR2 part of F5 might be involved in binding to the peptide’s C-terminal region. In addition, FR2 and CDR3 of F5 might bind to the HLA region around the peptide binding groove (Figure 3D). However, G9 prediction failed to show a similar binding pattern to F5, and it was instead predicted to bind HLA molecules only (data not shown).

### Cytotoxicity of F5- and G9-based CAR-T cells *in vitro*

**Figure 4:**
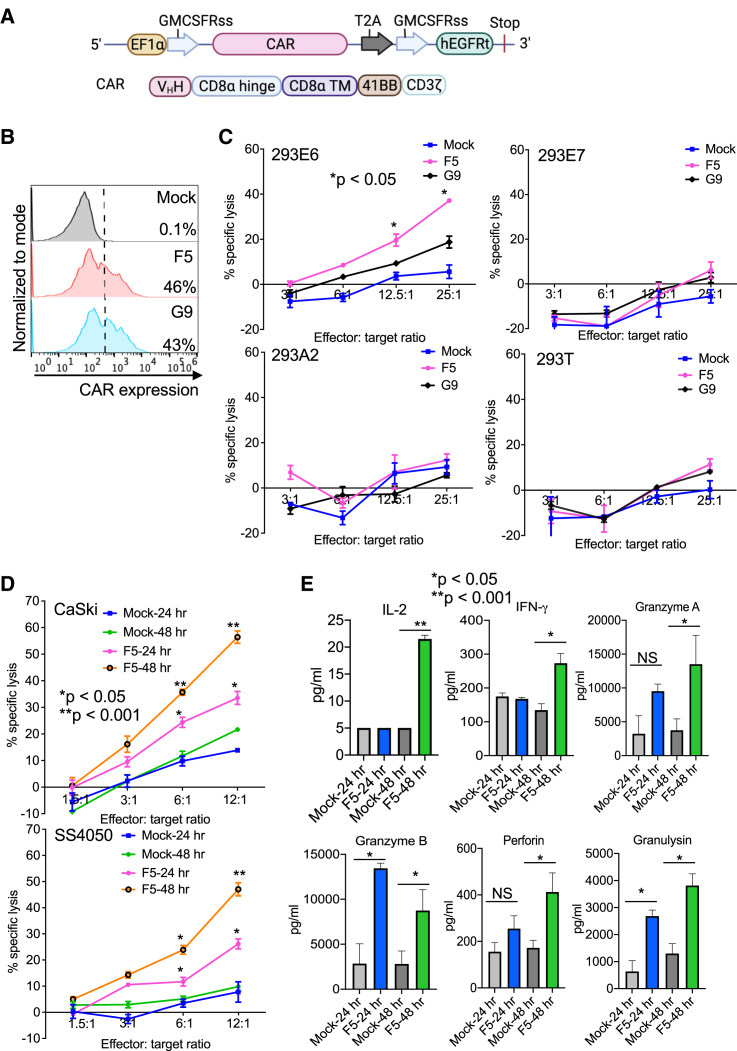
F5-based CAR-T cells showed specific and efficient cytotoxicity against target cells *in vitro* (A) Schematic design of CAR construction. (B) Transduction efficiency of F5 and G9 CAR-T cells produced. (C) F5 or G9 CAR-T cells were cocultured with target cells 293T, 293A2, 293E6, and 293E7 at different E/T ratios (3:1, 6:1, 12.5:1, and 25:1) for 24 h. The cell viability was measured by luciferase activity. (D) F5 CAR-T cells were cocultured with target cell CaSki or SS4050 at different E/T ratios (1.5:1, 3:1, 6:1, and 12:1) for 24 or 48 h. The cell viability was measured by luciferase activity. (E) The supernatant from CaSki and CAR-T coculture at an E/T ratio of 12:1 was collected and analyzed by multiplex cytokine assays on FACS. ∗*p* < 0.05 and ∗∗*p* < 0.001.

To demonstrate whether F5 and G9 can be used therapeutically for CAR-T treatment, we constructed the CAR containing F5 or G9 as the antigen recognition region, along with 4-1BB and CD3ζ signaling domains and a truncated human epidermal growth factor receptor (EGFR) cassette to gauge the transduction efficiency and inactivate CAR (Figure 4A). The transduction efficiency of F5 and G9 CAR-T cells was close to 50% (Figure 4B). Target cells, including 293E6, 293E7, 293A2, 293T, CaSki, and SS4050, were transduced with lentiviral GFP/luciferase and used for a luciferase-based cytolytic assay. Both mock T and CAR (F5 and G9) T cells were incubated with target cells for 24 h. As shown in Figure 4C, 293E6 cells were specifically lysed by F5 CAR-T cells in a dose-dependent manner compared with mock T cells. The killing ability of G9 was much weaker than F5, and thus it was excluded in the following studies. F5 CAR-T cells were incubated with CaSki or SS4050 cells at different effector/target (E/T) cell ratios for 24 or 48 h, and a robust killing was observed with E/T ratios of 12.5:1 and 6:1 (Figure 4D). The cytokine analysis of supernatant from the CaSki killing assay showed significantly higher levels of interleukin (IL)-2, interferon (IFN)-γ, granzymes A and B, perforin, and granulysin, which were released from CAR-T cells when cocultured with tumor cells for 24 or 48 h at 12:1 E/T ratios, while minimal cytokine production was observed from mock T cells (Figure 4F). Taking these results together, we concluded that F5 CAR-T cells could specifically lyse E6_29-38_ complex-positive human tumor cells.

### Activation of NFAT and NF-κB by F5-based CAR

**Figure 5:**
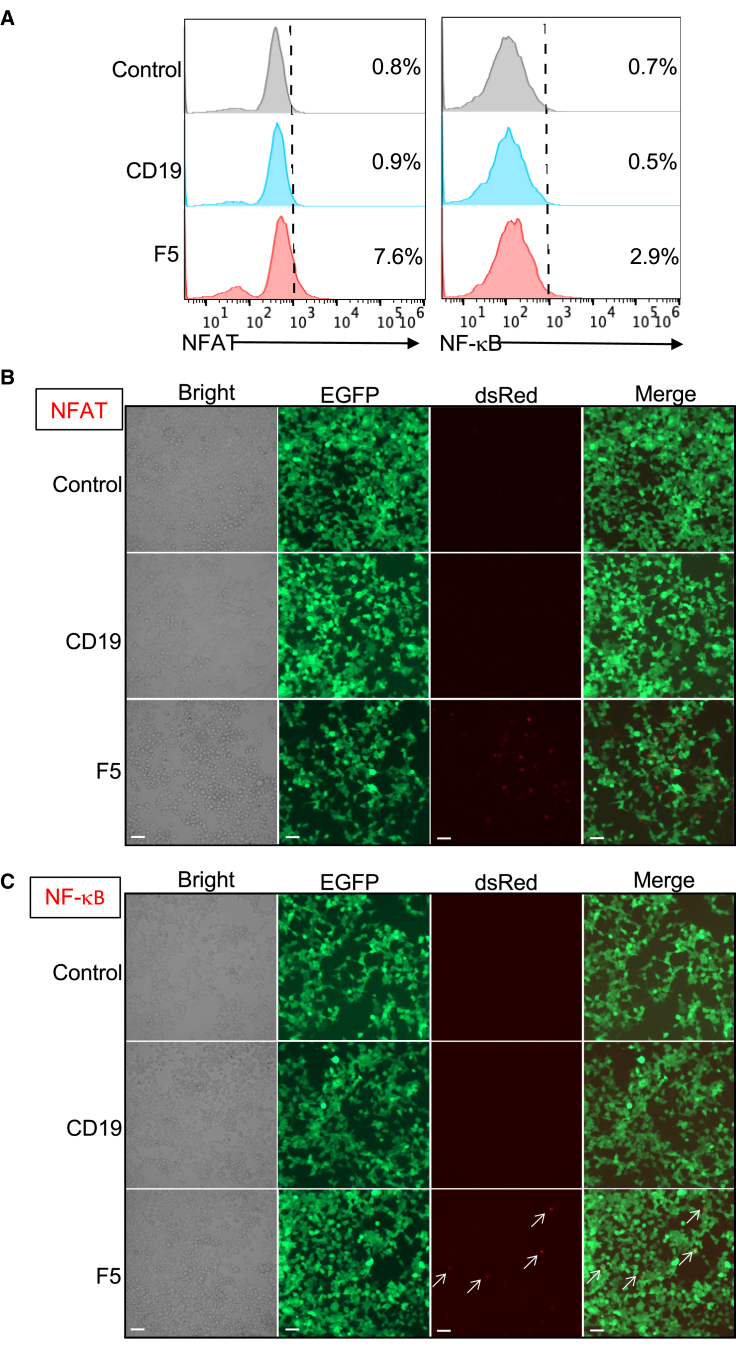
Both NFAT and NF-κB are involved in F5-based CAR-T activation (A) The Jurkat-CAR NFAT or NF-κB reporter cells after coculture with CaSki for 24 h were harvested and analyzed by flow cytometry and FlowJo. (B and C) The Jurkat-CAR NFAT or NF-κB reporter cells after coculture with CaSki for 24 h were imaged at 20× magnification under the microscope. Scale bar, 50 μm.

CAR stimulation leads to the phosphorylation of immunoreceptor tyrosine-base activation motifs (ITAMs) of the CAR CD3ζ domain and eventually activates TCR signaling pathways by inducing two transcription factors, nuclear factor of activated T cells (NFAT) and nuclear factor κB (NF-κB).^31^^,^^32^ To understand how F5 CAR-T cells act in the transcriptional level, we established Jurkat-CAR NFAT and NF-κB reporter cell lines that produce an enhanced tdTomato response to antigen stimulation following a previously established strategy.^33^^,^^34^ In coculture with an antigen expressing CaSki cell line, Jurkat-F5 NFAT or NF-κB reporter cells showed higher tdTomato expression compared with control groups, as observed by either flow cytometry (Figure 5A) or microscopy imaging (Figures 5B and 5C), indicating the involvement of F5-based CAR-T activation and cell killing.

### Inhibition of the CaSki xenograft growth by F5-based CAR-T cells

**Figure 6:**
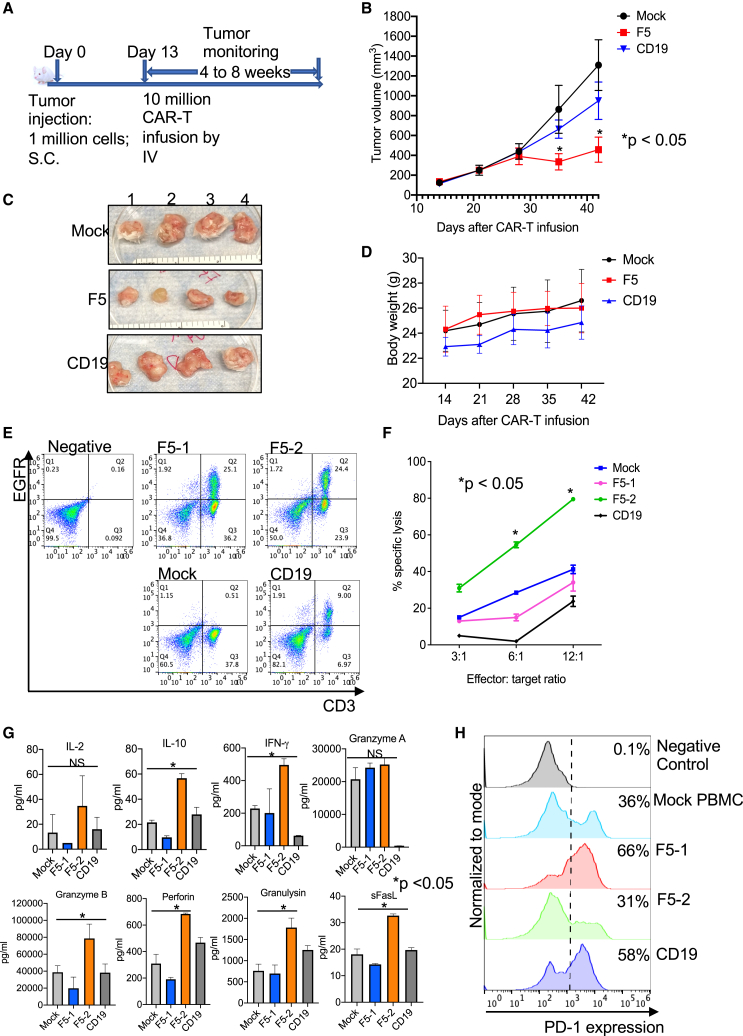
F5 CAR-T cells inhibited tumor growth in the CaSki xenograft model (A) Scheme of tumor inoculation, CAR-T injection, and tumor monitoring. (B) Tumor growth curve by caliper. (C) Tumor images before the final point. (D) Body weight during the process. (E) The spleens of the mice treated were harvested and cultured under IL-7, IL-15, and IL-21 for expansion of CAR-T cells. The cells were stained for EGFR and CD3 by FACS. (F) The expanded CAR-T cells were cocultured with CaSki cells at different E/T ratios for 24 h, and the cell viability was measured by luciferase activity. (G) The supernatant from CaSki and CAR-T cocultured at an E/T ratio of 12:1 was collected and analyzed by multiplex cytokine assays on FACS. (H) The cells were stained with anti-PD-1 antibody by FACS. ∗*p* < 0.05 and ∗∗*p* < 0.001.

To evaluate the antitumor efficacy of F5 CAR-T cells in mice, we established an HPV16+ cervical cancer xenograft model by subcutaneously injecting 1 million CaSki luciferase-expressing cells into the flank area of the mice. Fourteen days after tumor inoculation, mice were intravenously infused with either F5 CAR-T cells, antigen-mismatched CD19 CAR-T cells, or mock cells. The tumor volumes were monitored up to 7 weeks after CAR-T cell infusion (Figure 6A). As shown in Figures 6B–6D, F5 CAR-T cells reduced the tumor burden without a marked loss of body weight compared with the mock and CD19 CAR-T controls. The efficacy of tumor inhibition in the F5 CAR-T-treated group was not uniform among the mice, suggesting the heterogenicity of the xenograft tumor and uneven responses of CAR-T treatment. To determine CAR-T persistence, we recovered CAR-T cells from the mouse spleen. We found that *ex vivo* F5 CAR-T cells recovered from mice had a comparable persistence 6 weeks after infusion (Figure 6E). The spleen-isolated F5 CAR-T cells from the number 2 mouse (F5-2) still showed significant *ex vivo* cytotoxicity against CaSki cells, while the number 1 mouse (F5-1) did not, compared to control and CD19 cells (Figure 6F). The cytotoxicity of F5-2 was well correlated with higher cytokines release, including IL-2, IFN-γ, granzyme B, perforin, and granulysin (Figure 6G), compared with others. In addition, cells from F5-1 showed higher PD-1 expression than F5-2 cells, which suggested that, unlike F5-2 cells, *ex vivo* F5-1 cells had been exhausted and thus could not kill CaSki cells efficiently (Figure 6H).

### Inhibition of the SS4050 xenograft growth by F5-based CAR-T cells

We further tested the efficacy of F5 CAR-T in an additional cervical cancer xenograft model using SS4050 cells. SS4050 xenograft has more aggressive growth in mice than CaSki, and it will reach the tumor endpoint in 3 or 4 weeks (tumor volume greater than 2 cm^3^). On day 0, mice were injected subcutaneously with 1 million SS4050 cells. On day 7, mice were mock infused or infused with 10 million F5 CAR-T cells or CD19 CAR-T cells, and tumors were monitored for up to 3 weeks (Figure S4A). Mice treated with F5 CAR-T cells had smaller tumors than mock-treated and CD19 CAR-T cell-treated animals (Figures S4B and S4C). However, the efficacy of F5 CAR-T for SS4050 xenograft was worse than for CaSki xenograft. The spleens of treated mice were harvested on day 30 and cultured in media containing IL-7, IL-15, and IL-21 to allow for the expansion of CAR-T cells. Expression of EGFR and CD3 was measured by flow cytometry after 4 days of culture (Figure S4D). In addition, the expanded CAR-T cells were cocultured with SS4050 cells at different E/T ratios for 24 h, and cell viability was measured by luciferase activity (Figure S4E). The spleen-isolated F5 CAR-T cells from number 1 and 2 mice (F5-1 and F5-2) still showed *ex vivo* cytotoxicity against SS4050 cells, while number 7 mouse (F5-7) did not, compared to the control and CD19 groups. F5-7 isolated cells showed higher PD-1 expression than the other groups (Figure S4F). Together with Figure 6H, these data, collected from a total of 5 individual mice treated with F5 CAR-T cells in two independent experiments, suggest a potential correlation between CAR-T therapeutic efficacy and cell exhaustion as indicated by PD-1 expression. In addition, SS4050 cells had higher expression of PD-L1 than CaSki cells did (Figure S4G), which might contribute to the lower efficacy of F5 CAR-T in SS4050 models.

## Discussion

TCRm antibodies have been attractive for cell-based cancer immunotherapy for decades. However, isolating such antibodies has been challenging due to the buried peptide in a highly complicated and unpredictable MHC complex structure. Conventional antibodies may not efficiently reach the buried peptide. As a result, only a few TCRm antibodies are available in the field of cancer immunotherapy. Nanobodies might reach the buried site in the protein complex due to their small size and special loop structures interacting with the antigen. In the present study, we isolated and evaluated novel TCRm nanobodies that target an epitope of the oncogenic viral antigen HPV E6_29-38_ in the context of HLA-A∗02:01 and demonstrated therapeutic efficacy in models of HPV+ cervical cancer. This work may contribute to the field of cell-based immunotherapy in the following four aspects: (1) providing the feasibility to use a camel V_H_H nanobody as a therapeutic scaffold targeting MHC-associated peptides in cancer, (2) constructing large dromedary camel V_H_H phage libraries for the discovery of TCRm nanobodies, (3) targeting an MHC-associated peptide in governing optimal CAR-T activity without prior knowledge of the complex structure, and (4) inhibiting solid tumor growth in mice with TCRm nanobody-based CAR-T cells while the MHC-associated peptide density on tumor cells is low. This study has experimentally established F5 nanobody-based CAR-T as a promising new agent for treating cervical cancer and other HPV+ solid tumors and is ready for a first-in-human clinical trial.

Single-domain antibodies are small and can be used for modular building blocks for multi-domain constructs, antibody-drug conjugates, immunotoxins, or CAR therapy.^20^^,^^21^^,^^22^ Here, the F5 nanobody was converted into the CAR format. F5 CAR-T cells killed the cervical tumor cells *in vitro* and *in vivo* by releasing key cytolytic cytokines. Interestingly, it seems that F5 CAR-T has similar cytotoxicity efficacy *in vitro* using CaSki cells (specific lysis ∼30% at E/T of 12.5:1) to the E6-specific TCR gene-engineered T cells (specific lysis ∼30% at E/T of 50:1).^12^ Although the efficacy in different studies might not be comparable, F5 CAR-T has the clinical potential to treat HPV-related cervical cancer and other solid cancers lacking advanced cell therapeutics. Although F5 is derived from camel, camel nanobodies display high sequence similarity (∼75%–90%) with human VH (VH3 gene family) and are considered to have low immunogenicity.^35^ Nanobodies can be readily humanized and have appeared safe, with low immunogenicity, in recent clinical trials.^35^^,^^36^ It is of great interest to humanize F5 nanobody for clinical applications.

For CAR-T therapy in solid tumors, a combined strategy is essential to overcome tumor escape mechanisms and enhance the antitumor effect of CAR-T cells.^37^ CAR-T cells might need to be combined with monoclonal antibodies, small molecules, or bispecific CAR-T cells targeting different tumor-specific antigens.^37^ We observed a high PD-1 expression level in mice of the F5-treated group, indicating T cell exhaustion. To improve the efficacy, a combination of F5 CAR-T with PD-1/PD-L1 inhibitors might have a synergistic effect since immune checkpoint blockade could block PD-1 and PD-L1 signal activation, allowing CAR-T cells to kill cancer cells. We plan to test and optimize the combination of CAR-T therapy with anti-PD-1 or anti-PD-L1 antibodies in future experiments. HPV16+ tumor cells tend to have both E6 and E7 complexes presented on the cell surface.^14^ If E7 binders can be isolated, then a bispecific CAR targeting both E6 and E7 complexes might have substantial effects against the tumor.

HLA restriction is a significant drawback of TCR cell therapy. The development of TCRm antibodies that recognize the peptide across multiple HLA alleles might break the HLA restriction, which would benefit more populations of patients. There was a successful strategy to develop peptide-centric and single-chain antibody variable fragment (scFv)-based CARs that recognize the peptide presented by two HLAs, HLA-A∗23:01 and HLA-B∗14:02, in neuroblastoma.^38^ We hoped that F5 could be peptide-centric antibodies. Figure 1G showed that F5 and G9 had some binding to E7-MHC control, which also has HLA-A∗02:01, same as E6-MHC, implying that F5 and G9 could bind partially to HLA-A∗02:01. In addition, we tested the binding of F5 to the E6 peptide loaded with other HLA alleles including HLA-A∗11:01, HLA-A∗24:02, and HLA-A∗26:01 and found that F5 did not show obvious specific binding to those alleles other than HLA-A∗02:01(Figure S2). From the alanine scanning of the E6 peptide, we were able to speculate that F5 nanobody bound to the C-terminal part of the E6 peptide. The docking results of F5 with E6-MHC revealed that FR2 of F5 might bind to the E6 peptide C terminus. This should not represent genuine binding because it is not a structural binding study. It will be exciting and essential to structurally examine F5 binding to the complex through cryoelectron microscopy (cryo-EM) study. If the F5 binding region on HLA-A∗02 is conserved among HLA alleles from the cryo-EM study, then F5 might recognize the E6 peptide crossing different HLA subtypes.

In summary, we showed that F5 nanobody-based CAR-T cells can kill the cervical cancer cell lines *in vitro*, *in vivo*, and *ex vivo*. These findings demonstrated the feasibility and efficacy of CAR-T cells in targeting the intracellular E6 through the E6_29-38_ complex in the solid HPV16+-related tumor. In addition to the combination of PD-1/PD-L1 blockade and HPV E6/E7 targeting, future efforts should also focus on developing cross-HLA antibodies to benefit a broad range of cancer patients.

## Materials and methods

### Cells and reagents

Cell lines were cultured in media consisting of RPMI 1640 (CaSki, T2) or DMEM (SS4050, 293 lines) supplemented with 10% FBS and 1% penicillin-streptomycin at 37°C in a humidified atmosphere with 5% CO_2_. CaSki, SS4050, SCC90, and 293 lines were obtained from Dr. Christian Hinrich’s lab (NCI). CaSki, SS4050, and SCC90 cells are HLA-A∗02:01+ HPV16+ cervical cancer cell lines. The 293E6, 293E7, and 293A2 lines are 293-based lines with stable expression of HLA-A2 and E6, E7, or only HLA-A2, respectively. T2 cells were obtained from Dr. Jeffrey Schlom (NCI). Peripheral blood mononuclear cells (PBMCs) were isolated from the blood of healthy donors using Ficoll (Cytiva) according to the manufacturer’s instructions. All cell lines were authenticated by morphology and growth rate and were mycoplasma free.

### Peptides and monomers

Peptides were synthesized at a purity of >95% (Genscript, NJ). HLA-A∗02:01 and beta-2 microglobulin (β2m) were expressed and purified from bacteria separately. These two molecules were mixed with synthesized peptides (E6 or E7; purity >95%) and folded to generate a peptide/MHC complex (monomer): pMHC-E6, HLA-A∗02:01-TIHDIILECV; pMHC-E7, HLA-A∗02:01-YMLDLQPET. HLA-A2 was refolded with the peptide and β2m, purified by gel filtration, and biotinylated (NIH Tetramer Core at Emory University). The monomers were aliquoted and stored at −80°C for further applications. Additionally, monomers including HLA-A∗02:01, HLA-A∗11:01, and HLA-A∗24:02 were purchased from, while HLA-A∗26:01 was custom-made by, Kactus Bio. According to the company’s instructions, these monomers were mixed with the peptide at a molar ratio 1:10 for 30 min at room temperature to generate peptide/MHC complexes.

### Phage panning and ELISA

Camel single-domain antibody phage display libraries with a diversity greater than 10^10^ phage particles per milliliter were used for phage panning, as described previously.^39^ Briefly, the monomer (5 μg/mL) in PBS was coated on the immunotube at 4°C overnight. The immunotube or the 10^12^ phages were blocked with 3% skimmed milk in PBS/Tween 20 (0.05%) for 1 h at room temperature. Then, pre-blocked phage supernatant was added to the tube to allow binding. After 1 h of incubation at room temperature, the unbound and nonspecifically bound phages were removed using 10 washes with PBS/Tween 20 (0.05%) and 10 washes with PBS. The specifically bound phage was eluted with 500 μL of 100 mM trimethylaminade for 15 min at room temperature. The eluate was neutralized with 250 μL of 1 M Tris-HCl buffer (pH 7.5) and used to infect freshly prepared *E. coli* TG1 cells.

After four rounds of panning, 96 randomly picked clones were analyzed for antigen binding by monoclonal phage ELISA. Maxisorp 96-well plate (Fisher Scientific) was coated with the E6 complex or the control E7 complex. Phage ELISA followed previous protocols,^40^^,^^41^ and results were read using a spectrophotometer (Molecular Devices) at 450 nm.

### Antibody production and purification

The soluble antibody protein was produced and purified as previously described.^42^ Briefly, the pComb3x phagemids containing F5 or G9 sequences were transformed into HB2151 *E. coli* cells. The colonies were pooled and shaken in 1 L 2YT media containing 2% glucose and 100 μg/mL ampicillin at 37°C until the OD600 reached 0.8 to 1. Fresh 2YT media containing 1 mM IPTG (Sigma) and 100 μg/mL ampicillin were added after the bacteria cells were spun down. The culture was shaken at 30°C overnight for soluble protein production. The bacteria cells were spun down and lysed with polymyxin B (Sigma) for 1 h at 37°C to release the soluble protein. The supernatant was harvested after lysis and purified using the HisTrap column (Cytiva) using AKTA (GE Healthcare).

### Affinity measurement by Octet

The binding kinetics was determined using the Octet RED96 system (FortéBio) at the Biophysics Core at NHLBI, NIH (Bethesda, MD). F5 or G9 was immobilized onto NTA sensor tips. The antibody-coated tips were then dipped into PBS to stabilize the curve, into 25 nM E6 or E7 monomer for association, and then again into PBS for dissociation. Raw data were processed using Octet Data Analysis Software 9.0 to determine the K_D_ value.

### Flow cytometry

The binding of F5 and G9 to the E6-MHC complex on the cell surface was detected by anti-FLAG-APC-conjugated antibody. The transduction efficiencies of F5 CAR on T cells were detected by anti-EGFR human monoclonal antibody cetuximab (Erbitux) and goat-anti-human IgG-PE or allophycocyanin-conjugated antibody (Jackson ImmunoResearch). Data acquisition was performed using Sony A3800 (Sony) and analyzed using FlowJo software (TreeStar, Ashland).

### T2 peptide pulsing assay

One million T2 cells were pulsed with the peptides at a concentration of 50 μM overnight at 37°C. Cells were then stained with either an anti-HLA antibody (BB21, Invitrogen) to measure the expression of the complex or the nanobodies F5 or G9 and then anti-FLAG-APC for the antibody binding. Samples were acquired using a Sony A3800 flow cytometer. Flow cytometry data files were analyzed with the FlowJo software.

### CAR-T production and cell killing

V_H_H F5 was subcloned into the second-generation (2G) CAR construct, which contains expressing cassettes encoding the CD8α hinge and transmembrane region, a 4-1BB costimulatory domain, the intracellular CD3ζ, the self-cleaving T2A sequence, and the truncated human EGFR for cell tracking and ablation. The truncated human EGFR lacks the domains essential for ligand binding and tyrosine kinase activity but retains the binding epitope of the anti-EGFR monoclonal antibody cetuximab. Recombinant F5-CAR lentiviral vectors were produced by co-transfecting with packaging plasmid psPAX2 and enveloping plasmid pMD2.G into HEK-293T cells using CalFectin (SignaGen, Rockville, MD). Both psPAX2 and pMD2.G plasmids were gifts from Dr. Didier Trono (Addgene #12260 and #12259). Lentiviral particles were collected from the supernatant 72 h post-transfection and concentrated 100-fold by Lenti-X concentrator (Clontech, Mountain View, CA) following the manufacturer’s instructions. PBMCs from healthy donors were stimulated for 24 h using anti-CD3/anti-CD28 antibody-coated beads (Invitrogen, Carlsbad, CA) at a bead/cell ratio of 2:1, according to manufacturer’s instructions, in the presence of IL-2. To track T cell numbers over time, viable cells were counted using trypan blue.

The cytolytic activity of T cells transduced with F5-CAR was determined by a luciferase-based assay as described previously.^43^ Briefly, CAR T cells and luciferase-expressing target cells (293 lines and CaSki) were incubated for 24 h at different E:T ratios. The luciferase activity was measured using the luciferase assay system (Promega, Madison, WI) on a plate reader (PerkinElmer). The killing activity was normalized using mock T cells.

### NFAT/NF-κB fluorescence reporter assay and confocal microscopy

Following previously described methods,^33^^,^^34^ Jurkat-NFAT or Jurkat-NF-κB reporter cells were transduced with CAR-containing lentiviruses (F5; control CD19) at a multiplicity of infection (MOI) of 5. After determination of transduction efficiency, the reporter cells were co-incubated with GFP-overexpressed CaSki tumor cells at an E/T ratio of 1:1 in poly-L-lysine-coated μ-Slide for 24 h and then subjected to imaging using a Zeiss confocal microscope. In addition, the tdTomato expression level after co-incubation was measured using flow cytometry and quantified by FlowJo software.

### Animal studies

Five-week-old female NOD scid gamma (NSG) mice (NCI CCR Animal Resource Program/NCI Biological Testing Branch) were housed and treated under protocol LMB-059 approved by the Institutional Animal Care and Use Committee at the NIH. Tumors were initiated by subcutaneous injection of 1 × 10^6^ CaSki or SS4050 tumor cells on the flank. At 13 days post-tumor cell injection, tumor treatment consisting of a single intravenous infusion of T cells (either 10 million CAR-T cells or untransduced T cells) was done. Tumor volume (V) was determined by caliper measurement of tumor length (L) and width (W) and calculated with the formula V = (L × W × W)/2.

### Multiplex cytokine analysis

A human CD8/NK panel premixed bead-based multiplex cytokine assay from BioLegend can simultaneously detect the following cytokines by FACS: IL-2, IL-4, IL-6, IL-10, IL-17A, tumor necrosis factor alpha (TNF-α), sFas, sFasL, IFN-γ, granzyme A, granzyme B, perforin, and granulysin. Supernatants were collected from cultures that were assigned for functional studies and stored at −80°C until they were used for FACS. The data were analyzed according to the manufacturer’s manual.

### Statistical analysis

Statistical analyses were performed using Prism GraphPad software. For studies comparing two groups, a two-tailed unpaired Student’s t test was used. A *p* value of <0.05 was considered statistically significant.

## Data and code availability

The data generated in this study are available within the article and its supplemental information.

## Acknowledgments

This research was supported by the Intramural Research Program of 10.13039/100000002NIH, NCI Center for Cancer Research (CCR) Antibody Engineering Program (ZIC BC 011891 to M.H.). The dromedary camel V_H_H phage library construction was supported by the NIH Deputy Director for Intramural Research (DDIR) Innovation Award (to M.H.). The E6 and E7 monomers were obtained through the NIH Tetramer Core Facility. The MR1 tetramer technology was developed jointly by Dr. James McCluskey, Dr. Jamie Rossjohn, and Dr. David Fairlie, with the material produced by the NIH Tetramer Core Facility and permitted to be distributed by the University of Melbourne. The NIH Tetramer Facility is supported by contract 75N93020D00005 from the 10.13039/100000060National Institute of Allergy and Infectious Diseases, a component of NIH in the Department of Health and Human Services, United States. We thank the following researchers for their advice and technical assistance in growing T2 cells and setting up the T2 peptide pulsing assay: Dr. Jeffrey Schlom (NCI), Dr. James Gulley (NCI), Dr. Renee Donahue (NCI), Dr. Duane Hamilton (NCI), Dr. Jacqueline Douglass and Dr. Shibin Zhou (Johns Hopkins University), and Dr. Zhiyuan Yang (Eureka Therapeutics). We also thank Dr. Ling Zhang (NCI) for her assistance in growing SS4050 cells. We thank the NCI CCR Animal Resource Program/NCI Biological ranch for providing the NSG mice used in this study, the NCI CCR/Leidos Animal Facility for animal support, the NCI CCR Flow Cytometry Core Facility for assistance in cellular staining, the NIH Fellows Editorial Board for manuscript editing services, and the NCI CCR Genomics core for Sanger sequencing services. The content of this publication does not necessarily reflect the views or policies of the Department of Health and Human Services, nor does mention of trade names, commercial products, or organizations imply endorsement by the US government. The anti-HPV E6/E7 camel V_H_H single-domain antibodies such as F5 presented in this study are the subject of pending patent applications assigned to the NIH and are available for license in certain fields of use to qualified candidates. Please contact the corresponding author M.H. (NCI) at homi@mail.nih.gov if you are interested in pursuing a license. Cartoons in Figures 1A and 1B were created with BioRender.com.

## Author contributions

M.H., Z.D., and C.S.H. designed the project, oversaw the experiments, analyzed the data, and wrote the manuscript. D.L., N.L., S.L., and J.H. contributed to data analysis and editing of the manuscript. H.R. constructed the camel phage libraries. All authors read and approved the final version of the manuscript.

## Declaration of interests

M.H., Z.D., and C.S.H. are inventors on provisional patent application no. PCT/US2023/073144 entitled “Single domain antibodies targeting HPV E6/E7 oncogenic peptide/MHC complexes” assigned to the NIH. Inventors on NIH patents may receive blinded royalties from the NIH. C.S.H. is an advisor to and may receive compensation from GlaxoSmithKline, Neogene Therapeutics, PACT Pharma, and Capstan Therapeutics. C.S.H. receives research funding from Neogene Therapeutics and T Cure Biosciences. C.S.H receives in-kind support from Iovance Biotherapeutics and have equity and a position in Scarlet TCR. M.H. receives research funds from Lentigen/Miltenyi, CRISPR Therapeutics, Xyphos Biosciences, NeoImmuneTech, Cytovia Therapeutics, and Eureka Therapeutics via Cooperative Research and Development Agreements assigned to the NIH.

## Supplemental information

Document S1. Figures S1–S4 and Table S1

Document S2. Article plus supplemental information
