# Supplementary material for: CAR-T cells based on a TCR mimic nanobody targeting HPV16 E6 exhibit antitumor activity against cervical cancer
Source: Mol Ther Oncol. 2024 Oct 9;32(4):200892. doi: 10.1016/j.omton.2024.200892 (PMC11546159; doi:10.1016/j.omton.2024.200892)
Supplement: Document S1. Figures S1–S4 and Table S1 [file mmc1.pdf]

OMTON, Volume *32*

Supplemental information

CAR-T cells based on a TCR mimic nanobody targeting HPV16 E6 exhibit antitumor activity against cervical cancer

Zhijian Duan, Dan Li, Nan Li, Shaoli Lin, Hua Ren, Jessica Hong, Christian S. Hinrichs, and Mitchell Ho

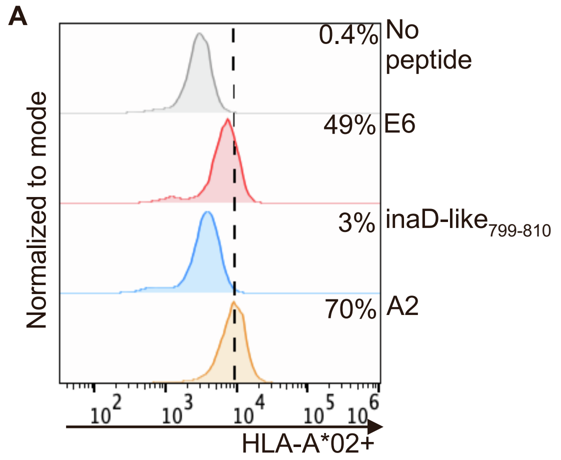

Figure S1. F5 has minimal binding activity to the peptide similar to E6$_{29\text{-}38}$. A,B) T2 cells were pulsed with the peptides (50 μM) indicated, and then examined by FACS for the expression of the complex and F5 binding at 5 μg/ml.

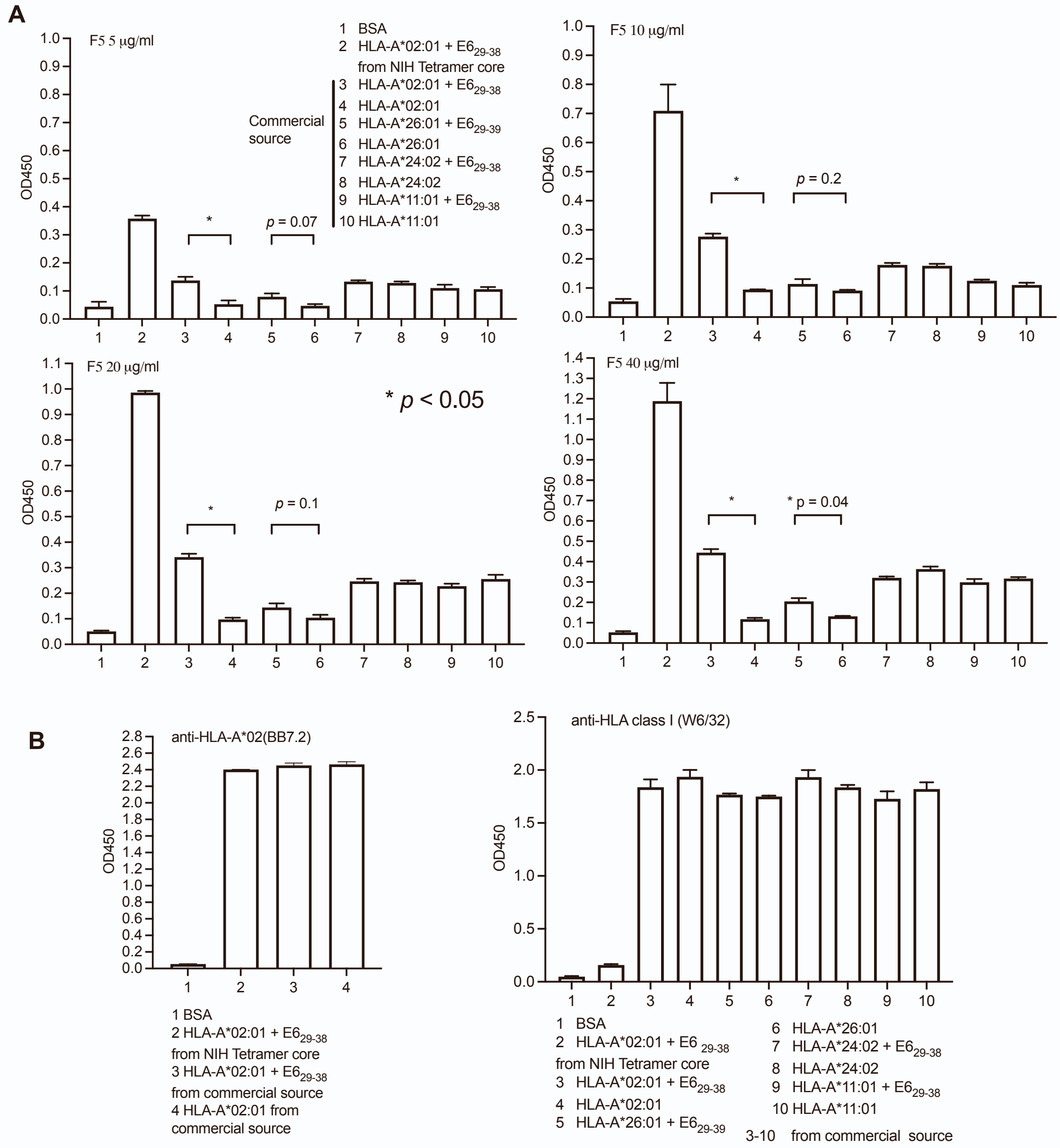

Figure S2. F5 binding to different monomers. According to the company's instructions, the E6 peptide was added to the monomer for at least 30 minutes at room temperature before use. Proteins (5 µg/ml) were coated on ELISA plate overnight in the refrigerator. Primary antibodies including F5 (A), anti-HLA-A*02 (BB7.2) and anti-HLA class I (W6/32) for HLA allele detection (B) were then incubated on the plate for 1 hour after blocking. HRP conjugated secondary antibodies were subsequently incubated for 1 hour. Results were read using a spectrophotometer (Molecular Devices) at 450 nm.

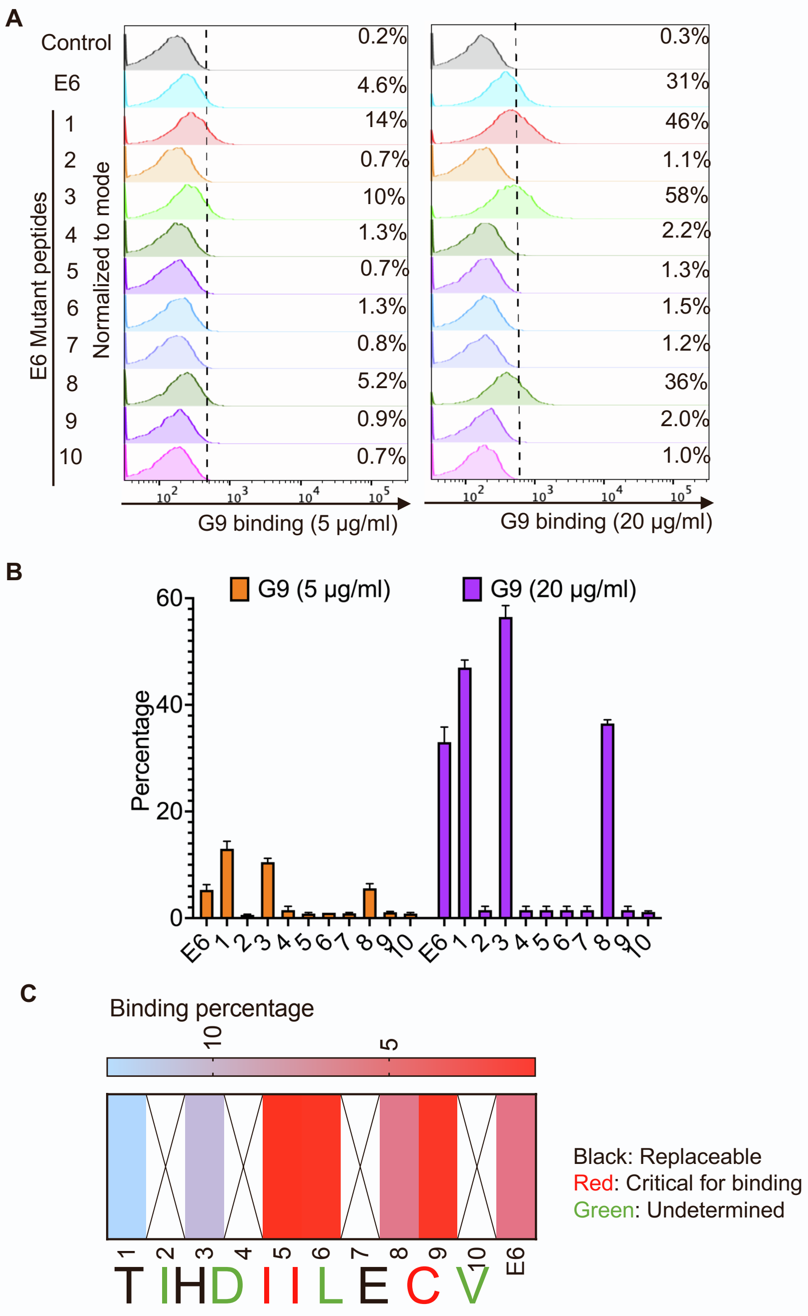

Figure S3. The C-terminal residues of E6 peptide were involved in the binding of G9 to the complex. A-C) T2 cells were pulsed with E6 and 10 mutated peptides, which have an individual single mutation to alanine at each position (50 µM), and then examined by FACS for the expression of the complex and G9 binding at 5 µg/ml and 20 µg/ml.

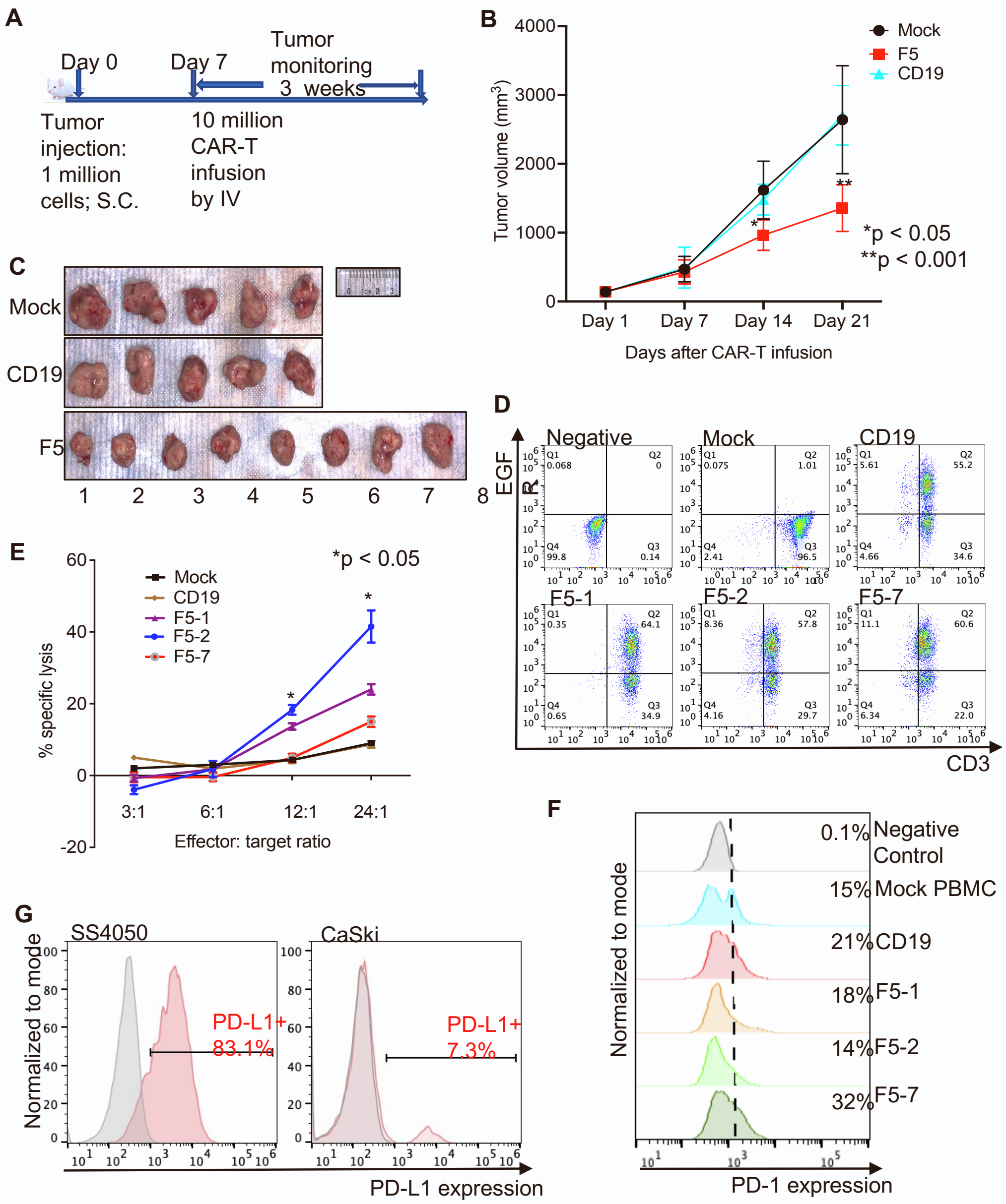

Figure S4. F5 CAR-T cells inhibited tumor growth in the SS4050 xenograft model. A) Scheme of tumor inoculation, CAR-T injection, and tumor monitoring. B) Tumor growth curve by caliper. C) Tumor images after the final point. D) The spleens of the mice treated were harvested and cultured under IL-7, IL-15, and IL-21 for expansion of CAR-T cells. The cells were stained for EGFR and CD3 by FACS. E) The expanded CAR-T cells were cocultured with SS4050 cells at different E/T ratios for 24 hours, and the cell viability was measured by luciferase activity. G) PD-L1 expression in SS4050 and CaSki was measured by FACS. F) The cells were stained with anti-PD-1 antibody by FACS. *p < 0.05; **p < 0.001.

Table S1 The sequences and purity of E6 and its mutant peptides

Peptide	Sequence	HPLC purity
E6	TIHDIILECV	96.2%
1	AIHDIILECV	97.1%
2	TAHDIILECV	95.9%
3	TIADIILECV	98.0%
4	TIHAIILECV	97.9%
5	TIHDAILECV	96.2%
6	TIHDIALECV	95.8%
7	TIHDIIAECV	96.2%
8	TIHDIILACV	95.1%
9	TIHDIILEAV	98.5%
10	TIHDIILECA	97.7%

Red: alanine substitution
